# Empathy, engagement, entrainment: the interaction dynamics of aesthetic experience

**DOI:** 10.1007/s10339-017-0805-x

**Published:** 2017-04-08

**Authors:** Ingar Brinck

**Affiliations:** Department of philosophy and cognitive science, Lund University, Lund, Sweden

**Keywords:** Aesthetic experience, Art, Dynamic system, Emotion, Empathy, Engagement, Inner imitation, Movement, Neuroaesthetics, Participatory sense-making, Simulation

## Abstract

A recent version of the view that aesthetic experience is based in empathy as inner imitation explains aesthetic experience as the automatic simulation of actions, emotions, and bodily sensations depicted in an artwork by motor neurons in the brain. Criticizing the simulation theory for committing to an erroneous concept of empathy and failing to distinguish regular from aesthetic experiences of art, I advance an alternative, dynamic approach and claim that aesthetic experience is enacted and skillful, based in the recognition of others’ experiences as distinct from one’s own. In combining insights from mainly psychology, phenomenology, and cognitive science, the dynamic approach aims to explain the emergence of aesthetic experience in terms of the reciprocal interaction between viewer and artwork. I argue that aesthetic experience emerges by participatory sense-making and revolves around movement as a means for creating meaning. While entrainment merely plays a preparatory part in this, aesthetic engagement constitutes the phenomenological side of coupling to an artwork and provides the context for exploration, and eventually for moving, seeing, and feeling with art. I submit that aesthetic experience emerges from bodily and emotional engagement with works of art via the complementary processes of the perception–action and motion–emotion loops. The former involves the embodied visual exploration of an artwork in physical space, and progressively structures and organizes visual experience by way of perceptual feedback from body movements made in response to the artwork. The latter concerns the movement qualities and shapes of implicit and explicit bodily responses to an artwork that cue emotion and thereby modulate over-all affect and attitude. The two processes cause the viewer to bodily and emotionally move with and be moved by individual works of art, and consequently to recognize another psychological orientation than her own, which explains how art can cause feelings of insight or awe and disclose aspects of life that are unfamiliar or novel to the viewer.

## Feeling with works of art: empathy and aesthetic experience

Works of art tend to evoke strong experiences in the viewer. In engaging with a painting or sculpture you sometimes can feel that you are sharing experiences with it: You have the sensation of feeling with it, of empathizing. At other times, engaging with work of other artists, you distinctly know you are not sharing any experiences, but the experiences you nevertheless undergo genuinely appear to emanate from and belong to the artwork. How are such feelings of connectedness and estrangement with an artwork possible?

This issue may not be as strange as it may seem, because feeling with other people and feeling with works of art have affinities that suggest a common basis in empathy—the feeling of understanding others’ experiences and thoughts from their perspective. Empathy directly presents you with another subjective perspective on the world than your own, which does not seem to originate within you, but is encountered as a *fait accompli*. Furthermore, both aesthetic experience and the experience of another person’s subjective perspective have another qualitative profile than everyday experience and can be strongly moving. Ignoring functional and pragmatic properties, they are characterized by high arousal, sustained attention, and marked personal engagement both cognitively and emotionally (Markovic 2012; Vessel et al. 2012).

The feeling of understanding others’ subjective experiences typically arise while interacting with them in the second person. Taking the intuition that second-person and aesthetic experience share a common basis in empathy at face value, the present article examines the role of empathy for aesthetic experience from a theoretical, interdisciplinary perspective that merges insights from psychology, philosophy, phenomenology, and neuroscience within the dynamic framework. Claiming that aesthetic experience depends on moving with and being moved by the artwork, the aim is to explain aesthetic experience in terms of the processes that cause it to unfold by presenting a series of empirically well-grounded hypotheses about the relational dynamics between viewer and artwork.

Historically, following Immanuel Kant aesthetic experience has been interpreted as an intellectual feat, pleasing by its pure form. Marked by Kant’s distinction between disinterested judgements of taste and bodily judgements of sense, mainstream 19th and 20th century aesthetics ignored pre-reflective, non-conceptual, emotional, and bodily responses to art. The strong focus on propositional and discursive processes left other processes largely unexplored. Today there is wide-spread interest in the functions of emotion, perception, and bodily sensation for aesthetic experience and in empathy as its source (Crowther 1993; Dengerink Chaplin 2005; Freedberg 2012; Freedberg and Gallese 2007; Haworth 1997; Scarinzi 2015; Shusterman 2000). Although reflection and verbal interpretation may play a significant part in aesthetic experience, they are not necessary.

The view that aesthetic experience is based in empathy and occurs by the mental and bodily simulation of elements depicted in the artwork recently has been resurrected in the research on aesthetics in neurosciences. I will criticize both the original version of the simulation theory and its contemporary version in neuroaesthetics, and defend a dynamic approach to aesthetics that describes the perception of art and aesthetic experience as embodied, embedded, and enacted in engagement with the artwork. I suggest that aesthetic perception is explorative and involves intelligent perceptual and motor skills, while explaining the emergence of aesthetic experience by reference to the relational dynamics between viewer and artwork. I argue that viewer-artwork interaction can be modelled as participatory sense-making, proposing that entrainment creates an implicit common ground that constitutes the baseline for interaction. Drawing on research in developmental psychology on empathy, I submit that aesthetic experience emerges by bodily and emotional engagement with works of art via the complementary processes of the perception–action and motion–emotion loops. These processes enable the viewer to move with and be moved by art.

## The simulation theory of aesthetic experience: critical remarks

The view that aesthetic experience involves empathy was developed by Robert Vischer (1873). According to Vischer, aesthetic experience consists in the genuine empathy towards pure form, evidenced by the involuntary inclination to feel static form move freely and the spontaneous experience of a rhythmic continuity between self and artefact. Vischer stressed the importance of emotion and imagination for understanding art and argued that aesthetic experience results from *Einfühlung*, the act of feeling into the observed forms of works of art. The viewer places herself at the centre of gravity of the artwork and thinks her way into it. Then the imagination permits simulation of the initially vague contents of sensation as sensuous concrete form. Forms cause affects in the viewer that paired with the free association of ideas enable aesthetic appreciation. Wölfflin (1886) explained the aesthetic experience of architecture from a similar standpoint. Like Vischer, he held that empathy begins in bodily simulation and ends in mental simulation through the projection of first-person experiences into the physical forms of buildings and art.

Elements of Vischer’s theory recur in contemporary neuroaesthetics that investigates how aesthetic experience depends on the ability to identify with forms in pictorial content. In a study on dynamism perception, Massaro et al. (2012) compare the processing of pictorial content including human subjects with the processing of pictorial content including nature and show that dynamism plays a role in both cases. They explain dynamism perception concerning human content by reference to the bodily simulation of other agents’ actions, but cannot find a plausible physiological explanation of it concerning nature content. Because proprioception is implied in the processing by parieto-premotor sensory-motor circuits that send feedback to the visual areas in the brain, they venture that embodiment may be relevant. The notion of embodiment entails functional and constitutive dependency on implicit sensorimotor processes and bodily experience (Beer 2014; Clark 1997; Kirsh 1995; Varela et al. 1991; Wilson 2002). To elucidate how it might explain inanimate dynamism perception, Massaro and colleagues quote Wölfflin (1886:151):

Physical forms possess a character only because we ourselves possess a body. If we were purely visual beings, we would always be denied an aesthetic judgment of the physical world. But as human beings with a body that teaches us the nature of gravity, contraction, strength, and so on, we gather the experience that enables us to identify with the conditions of other forms.

Pursuing the research of Massaro and colleagues, Di Dio et al. (2016) conclude that in naïve subjects, human dynamic content causes motor resonance, while static nature content causes imaginary motor simulation that reflects the functional potential of represented landscapes.

In spite of its broad acceptance, Vischer’s theoretical framework faces difficulties that can be traced to his notion of empathy. Empathy is described as consisting in two consecutive processes: Mental states are first simulated with the body and then mentally projected into the object. I will clarify why this conception of empathy is problematic by reference to the notions of simulation and projection.

To begin, simulation entails mimicking the states or processes of the model, and successful simulation of another person’s experiences results in the literal sharing of her experiences. However, literal sharing stops short at reproducing what the other person feels, which means it ignores the gist of empathy. The function of empathy is the opposite, viz. to recognize another person’s experiences as his. Phenomenologically speaking, in empathy you are confronted with the presence of a qualitative experience that you are not living through yourself (Zahavi and Rochat 2015). The awareness of the other person’s experience as distinct from your own permits responding to it by reciprocating, e.g. comforting a person who is experiencing sadness, relieving her agony if she is experiencing pain, or rejoicing with her if she is happy (Zahavi 2008).

Turning to projection, it involves the transfer of experiences from self to other by analogy, placing oneself instead of the other person at the centre of the process. This procedure clearly conflicts with the reciprocal nature of empathy. Instead the accurate perception of another person’s experience requires recognizing its radical otherness. Interpreting others in terms of one’s own experiences and feelings complicates separating one’s own and others’ reactions and furthermore reduces the usefulness of empathy in clinical contexts of medical practice and psychotherapy (Halpern 2001, 2003). Hence, projection is not necessary, nor desirable for empathy. We understand others directly and non-inferentially by perceptual acquaintance with them as living bodies (Gallagher 2001, 2008; Krueger 2012; Scheler 1954; Zahavi 2008). We simply can see what they desire and need, fear and avoid, feel and intend. Briefly, empathy is based in the immediate recognition of another person’s experience as distinct from your own.

Freedberg and Gallese (2007; see also Freedberg 2012) have advanced a version of Vischer’s theory that explains aesthetic experience as the simulation of actions and emotions by mirror neurons in the brain. They argue that the observation of goal-directed action, artefacts via the actions they afford, bodily and facial expression of emotion, real or implied body movement, and traces of instrumental action (e.g. footsteps on the ground, pencil strokes on paper, or chisel marks on a sculpture) activates roughly the same neurons in the observer’s brain as in the agent’s and results in simulation of the corresponding motor action or emotion. Hence, the simulation or mirror mechanism in the brain is responsible for aesthetic experience.

To their advantage, Freedberg and Gallese can explain the directness of experience, the brain’s responses being automatic, and deny that empathy involves projection of the observer’s own emotional reactions. However, like Vischer they conceive of empathy as the sharing of experiences, which conflicts with the core function of empathy: reciprocity. Additionally, it is questionable that activity in the mirror neurons is adequate for explaining qualitatively felt aesthetic experience.

Freedberg and Gallese claim that the observation of a movement (or action, facial expression, gaze, etc.) in an artwork will cause simulation of the movement, similarly to how the observation of a real movement would cause simulation, and result in the experience of it. This raises the question why the resulting experience would amount to an aesthetic as opposed to regular experience of movement and emotion. Apparently the two types of experience occur by the same kind of operation. The obvious difference lies in their causal history, the one being caused by observation of a real movement, the other by observation of a movement in an artwork. However, there is no mention that the nature of the cause would influence the processing significantly; rather, the point of the theory is to provide the same explanation in both cases. Accordingly, Freedberg’s and Gallese’s hypothesis leaves it undetermined what makes an experience aesthetic.

To stress, denying that the simulation hypothesis provides a satisfactory explanation of aesthetic experience is not to deny that mirror neurons are involved in the causal realization of responses to art. Motor, somatosensory, and visceromotor processes are implicated in the visual processing of works of art; the uncertainty concerns their exact function for specifically aesthetic experience. One of the central aims of art is to make the viewer experience something unfamiliar or out of the ordinary. Sometimes this amounts to presenting figurative or non-figurative counter images or disclosing unknown aspects of the world and in doing so produce feelings of insight, learning, surprise, or awe. Like empathy, aesthetic experience depends on grasping the difference between one’s own experiences and such that have their origin in others. Acknowledging the radical otherness of those that originate in the artwork enables the psychological re-orientation that characterizes aesthetic experience.

Let us take stock. I have argued that the notions of simulation and projection lead in the wrong direction. Conceiving of aesthetic experience as a matter of motor simulation of elements depicted in the artwork is misguided, because the simulation mechanism does not distinguish aesthetically relevant information (in terms of valence and potential action) from socially and instrumentally relevant information.

## The nature of aesthetic perception: an acquired skill

Like everyday experience, aesthetic experience is enacted. Although it causally depends on the brain, it is not caused by and realized in the brain, but in the world by an embodied agent (Noë 2004: 227; cf. Smith 2005). Aesthetic experience arises in the active probing of a certain kind of material artefact in physical space, viz. the work of art.

Perception is adaptive: It has evolved to keep the organism in harmony with its niche and sustain its existence (Gibson 1986). It also is explorative: Responding to changes in the environment demands exploring new ways to exploit it (McGann, De Jaegher and Di Paolo 2013). The interdependence between adaptive and explorative behaviour explains why, when “nature” is transformed into art, reality sometimes appears more transparent than ever to the great satisfaction of both artist and viewer. Exploration improves transparency.

The skillful perception of artworks manifests a knowhow that develops over time and gradually increases the depth and complexity of aesthetic experience. The viewer learns how to see and act, what to attend to and how (Gibson 1986; Ingold 2001). Exploring works of art draws on similar implicit and attention-guided learning and non-representational (meta)cognitive abilities as other types of skillful bodily action, e.g. modern dance or figure skating (Brinck 1999). It is monitored and controlled independently of reflection, and its progress is continuously evaluated, not necessarily relative to a goal (exploration may be its own goal) but by its moment–moment quality, organization, variation, and deviation (Brinck and Liljenfors 2013). These processes form part of the over-all behaviour and can be phenomenologically and perceptually transparent, available to the agent on the personal level (Montero 2010; Toner, Montero and Moran 2015).

Phenomenologist Maurice Merleau-Ponty’s work on perception and art articulates a complementary outlook to enactivism. Focusing on its performative aspects, Merleau-Ponty (1964) describes the perception of art in the first person. In previous work (Brinck 2003, 2007) I have brought these theoretical perspectives together, and describe the production and consumption of art as contrasting, yet interrelated dimensions of a multi-directional practice that constitutively depends on the material and cultural properties of the environment.

Gibson’s (1986) notions of affordance and effectivity prove useful for explaining how artists and art lovers can share their different experiences of art and participate in joint practices by living in the same environment (Brinck 2003). An affordance is a functional property of an object that exists relative to an agent and defines the sum of possible actions that involve the object. Affordances simultaneously constrain and enable behaviour. An effectivity is a functional property of an agent that defines the agent’s operative skills relative to the affordance of an object in a given context. Because objects engage attention through the functional properties that correspond to the agent’s effectivities, an agent’s effectivities will shape her ways of interacting with the environment, granting her access to a limited set of affordances.

In making art, the aesthetic quality of the interaction emerges from the particular effectivities that allow the artist to access affordances that correspond to her personal style (Brinck 2007). Her operative skills will determine which information she will pick up when and how. Artists acquire their individual style, a certain manner of engaging with the context via sensorimotor processing, through the repeated physical activity of producing art (Merleau-Ponty 1964). The painter Edouard Pignon (1966) describes how an artist’s bodily experience of space and time conditions the forms and colours of her work. He maintains that artists develop aesthetic perception by gradually refining their technique, and that learning to perceive and act in a distinctive way takes years of practice. I suggest that, conversely, viewers can learn to recognize a particular artist’s style by familiarizing with the artist’s ways of handling the many aspects of common space—physical, temporal, material, social, cultural, and historical—by interacting with the artist’s work (Brinck 2003, 2007). Repeated encounters with art will cause viewers to develop skills for perceiving art that progressively changes the quality of their aesthetic experiences.

Learning takes place within socially and culturally circumscribed activities and involves the transfer of skills and traditions by artefacts, procedures, rituals, and narratives (Brown et al. 1989; Lave 1988). Knowledge is distributed, extended in space and time and continuous with processes in the environment (Hutchins 1995). Because external resources such as the technologies and commodities that support cognition change over time, processes of the same type, say, memorizing something, differ radically depending on the place and time when they occur—some 10,000 years ago, in the last century, or today (Donald 1991). This holds true for perceiving objects of art too.

Discussing how ship navigators use divider and scale to find the way, Hutchins (2010:433) asserts that what is seen is other than merely what is visible; it is “there by virtue of the activity of seeing being conducted in a particular way”. Thus, the practices of reading the span of the scale as speed or distance see something different in the very same visual array. Similarly, because aesthetic experience is enacted, or acted out, what viewers experience in engaging with works of art is determined by what they do, know how to do, and are ready to do (cf. Noë 2004:1f). Perceptual skills such as the ability to enact relationships among independent items and recognize patterns that go unnoticed or have to be calculated by less experienced subjects (Kellman and Garrigan 2009) go hand in hand with contextualized sensorimotor skills such as knowing how to practically engage with a certain artefact (cf. McGann et al. 2013).

Perceptual and sensorimotor skills play a decisive role for the quality of aesthetic experience and support direct, on-line understanding of artworks, much like they support social understanding and empathy. By way of example, consider the many physical traces that an artist’s movements and actions leave in the artwork and that witness the craft of making it. Observing them, the novice may be able to stepwise re-construct the creative process from a third-person perspective and gain some insight into it. In contrast, the skilled viewer knows what kind of information to look for and how to act when detecting it. She can construct the causal sequence from an involved second-person perspective and re-enact the artist’s bodily movements and gestures with some precision, gaining access to the artist’s way of seeing that gives the artist’s motor actions their personal signature (Brinck 2007). Merleau-Ponty elucidates the present line of thought from the perspective of phenomenology. He writes: “I can meet in things the actions of another and find in these actions a sense, because they are themes of possible activity for my own body” and “[I] find others at the point of origin of the actions [I] imitate” (Lawlor and Toadvine 2007:146).

To return to the discussion of brain simulation in the previous section, paying attention to the physical properties of works of art while enacting them promotes aesthetic experience in additional ways to those acknowledged by neuroaesthetics. The carvings and marks in the stone of a sculpture by chisel and hammer and the strokes of the brush and knife against the canvas of a painting give insights into the dynamics of the creative process and reveal the artist’s web of intentions, sensations, and feelings through their spatial, material, and physical properties such as direction, shape, quantity, location, relative size, grain, refinement, delicacy, and density. Embodied engagement with an artwork prepares for a phenomenologically richer understanding than the detached, observational perspective that informs the viewer about mainly her own reactions to the artwork.

The next section introduces the dynamic approach to aesthetic experience, which in subsequent sections will provide the tools for explaining the emergence of aesthetic experience.

## The dynamic approach to aesthetic experience: making sense of art

According to dynamic systems theory, agents interact with the physical environment by coupling to it, which entails that agent and environment mutually and continuously influence each other (Varela et al. 1991). Variations in agent and environment form patterns that improve the conditions for the interaction and serve to maintain it (Beer 2000; Thelen and Smith 1994). Cognition is set to preserve the autonomy and continued existence of dynamic systems by neutralizing external and internal perturbations. On the dynamic approach, cognition is a relational, historical process: What matters is not which internal states agents have, but what agents do (De Jaegher and Di Paolo 2007; Thompson and Stapleton 2009).

Enactivism presents a complementary account of cognition in terms of how sense-making regulates the interaction of coupled systems (De Jaegher and Di Paolo 2007; Froese and Di Paolo 2011; cf. Varela, Thompson and Rosch 1991). By coordinating with stimuli that have subjective value, agents can perceive and act on valences, subjective positive-to-negative evaluations of experiences, in the environment. Choosing the stimuli to which it will be sensitive permits the agent to enact a meaningful world that ensures its continued existence, and transforms the objective world into a place of salience and value that reflects the needs of the individual (Thompson and Stapleton 2009).

Importantly, in the dynamic framework, social understanding and empathy are based in coordination, i.e. patterned behaviour organized with respect to timing, rhythm, and (de)synchronization (Di Paolo et al. 2010). Understanding does not include de-coding or retrieval of representations, nor the matching or projection of emotional, perceptual, or intentional inner experiences (Hutto 2015). There is nothing “there” in the individual that waits to be shared. Experience and meaning do not as such exist before the interaction takes place, but are transitory phenomena that emerge in the process of sense-making.

Admittedly, focusing on the coordination patterns that organize the relational dynamics of coupled systems has limitations, because it ignores exogenous influences. It will explain how agents make sense of the world they inhabit by identifying the parameters that control their behaviour without considering the environmental effects. Most contextual properties of significance for cognition have been tuned to human agents by biological evolution and in a shorter historical perspective epistemic niche construction, and systematically influence perception and action (Barker 1968; Brinck 2009; Donald 1991; Heft 2007). Sense-making is embedded: functionally and constitutively dependent on temporal, material, technical, social, and cultural aspects of the environment. Haugeland (1998) describes the embeddedness of embodied agency in Heideggerian terms as the intimacy of the mind’s being in the world, characterized by an integralness of mind, body, and world that undermines their very distinctness.

Aesthetic experience is scaffolded by technology and material culture and socio-culturally by rituals, habits, norms, and scripts. While large societies often show great diversity in the expression of art, the artworks nevertheless are fundamentally interrelated, because they are grounded in the same material culture (Malafouris 2013). The integralness of mind, body, and world permit understanding how art can work its wonders. What individual agents can do and how they interact depend on what material, technological, and symbolic resources are available to them, and if and how they can access these resources (Brinck 2003). Given that artist and viewer are contemporary and take part in the same material culture, the interdependence between cognition and environment causes the artist to create art and the viewer to make sense of it in ways that intrinsically connect.

To repeat, as opposed to theories that explain cognition by the properties of the agents, the dynamic approach explains cognition in terms of the relational dynamics between the agents. The emphasis on relational instead of agent properties makes it possible to model agent-artefact interaction on agent-agent interaction. In the present context, doing so will have the advantage of representing the causal influence between viewer and artwork as bi-directional, and avoids downgrading the contribution of the artwork or exaggerating the viewer’s efforts as a mere effect of the explanatory framework. To the same end, I suggest conceiving of aesthetic experience as the result of participatory sense-making that generates significance by joint interaction (De Jaegher and Di Paolo 2007). This will reveal features of the interaction that unidirectional frameworks do not capture such as reciprocity.

Participatory sense-making is unavailable to single agents and cannot be reduced to patterned behaviour. Crucially, it involves *movement* as the manifestation of intentional activity (De Jaegher and Di Paolo 2007). One might object that because participatory sense-making is intentional, it is inadequate for explaining aesthetic experience, which involves interaction with an artefact. This point makes it a central task to examine, *first*, on what grounds movement can be ascribed to artefacts that are artworks, and *second*, what kinds of movement would enable the viewer to feel with the artwork and cause the emergence of aesthetic experience. The second task concerns movements of both viewer and artwork, and such that by regulating the dynamics are conducive to aesthetic experience.

Regarding the first issue, there is evidence that artworks can control viewers’ perception and shape their emotional response, and in that sense may be held to manifest intentional activity. Eye-tracking studies of how people look at artworks show that artworks act on viewers’ perception systematically and that viewers respond differentially. The studies reveal common patterns based in principles such as contrast, regularity, and saliency that drive the attention to particular areas, suggesting that gaze is guided by the artwork, but also reveal large variability depending on subjects’ interest, artistic appreciation, previous experience, and knowledge, which means that the effect of the artwork is not mandatory or predetermined (Quiroga and Pedreira 2011). For instance, experimental manipulations of paintings by Piet Mondrian concerning the orientation, proportional relations, and colours of the components have been shown to with certain regularity steer the attention to other areas than the original paintings do (ibid; Locher et al. 2005), whereas a series of studies of how the eyes actively explore a painting by Francis Bacon demonstrated significant difference between art-trained and nonart-trained participants, e.g. art-trained observers fixated regions important for spatial construction while nonart-trained observers ignored them (Kapoula and Lestocart 2006).

Paintings also influence viewers’ emotions in predictable ways. Melcher and Bacci (2013) found that there is a strong bottom-up and objective aspect to perception of emotion in abstract artworks that may tap into basic visual mechanisms, in that features such as colour, line, form, and composition reliably prime a certain emotion. van Paasschen et al. (2015) report that affective evaluations of art in terms of valence and arousal were consistent among observers in ratings for representational and abstract artworks, while judgments about beauty and wanting differ between experts and novices. This agrees with Silvia’s (2013) findings that knowledge emotions such as confusion and interest that are appraisals of high novelty and degree of comprehensibility have different weights for experts and novices. To stress, these results should not be taken to buttress the view that there are aesthetic primitives that determine the aesthetic value of a stimulus. Aesthetic preferences and judgments depend on a wide range of factors that may not be the same across contexts, similarly to interpersonal preferences and judgments.

The cited evidence shows that artworks exert significant influence on viewers, and that viewers’ reactions differ systematically. Does it grant ascribing movement to works of art? Movement is a self-sustained process from one position to another that has a forward direction towards something or somebody, and can fail or succeed. It creates difference by making new facets of the environment available to the agent that promise to meet her needs. Thus movement has meaning or subjective value because the difference it creates, makes a difference to the agent as an individual, be it positive or negative, minor or major. That artworks sometimes make a difference by revealing unknown aspects of human life or existence to the audience should be uncontroversial. Art is known to change the ways in which people perceive or feel. Consequently, it would seem part of the way art operates to influence or act on viewers’ perception, behaviour, and understanding, which means that artworks may be directed in the sense of targeting the viewer’s cognition and mind.

In all, the discussion suggests that artworks can exhibit movement and generally have the capacity to move and produce movement in viewers, even if this may not be true in every single case. I conclude that we are licensed to explain aesthetic experience by the participatory sense-making of viewer and artwork. It remains to deal with the second task, and determine what movements regulate the interaction dynamics and enable feeling with the artwork. This issue will be examined in “Moving together: the means for participatory sense-making” and “Bodily engagement: the perception-action loop” sections. The next section concerns entrainment where all interaction begins.

## Entrainment: the baseline

Entrainment is the ubiquitous tendency of physical and biological systems to coordinate to autonomous, spatially or temporally structured events or rhythmic movements, and involves the detection of and response to rhythm and the integration of the systems by synchronization (Clayton et al. 2004; Glass 2001; Phillips-Silver et al. 2010; Wilson and Wilson 2005). Rhythm consists in the patterned recurrence of a regular or irregular element, e.g. a beat, form, sound, or movement, in a temporal or spatial sequence. Repetition constitutes rhythm if it involves variations. There is great dissimilarity in the periodicity, timing, intensity, frequency, regularity, amplitude, and predictability of processes of entrainment. The coordination between biological systems typically is relative by phase attraction, moving into and out of the zone that surrounds perfect synchrony. The heartbeat, blood circulation, and respiration are naturally occurring rhythms.

Social entrainment between individuals is a special case of spatiotemporal dynamic coordination. It usually is implicit, subconscious, and automatic and causes mutually constraining, stabilizing behaviour by alignment and matching, e.g. motor mimicry when a speaker assumes the same accent or tone of voice as another speaker during conversation, movement coordination when two persons fall into the same pace while walking side by side or spontaneously make a certain gesture at the same time, and mirroring when people adopt one another’s body posture and orientation (Knoblich et al. 2011; Schmidt and Richardson 2008).

Because entrainment comes naturally and the inclination to entrain is strong and requires effort to control, people can be expected to synchronize to the rhythm of any stimulus, animate or not. Visitors to galleries and museums are likely to automatically entrain to the rhythm of any artwork that attracts the attention and match body or head orientation, posture, core affect, gaze, and/or state of arousal to it.

Rhythm is a well-known design principle in the visual arts of all times (Sayre 2015). In two-dimensional images such as regular paintings, lithographs, and drawings, rhythm is created by the recurrence of lines, dots, shapes, colour patches, strokes of the brush or pen or knife, and of figurative motives, e.g. a fish repeated at different positions or alongside with slight modifications, or children who perform the same action, say, running. The recurring juxtaposition of contrastive non-figurative elements also creates rhythm. To illustrate, Jackson Pollock made paint drip from a can onto canvases placed on the floor or against the wall and then used knives, trowels, and sticks to add depth to the images. This resulted in paintings that lack clear emphases and exhibit random rhythm. In contrast, Agnes Martin’s signature paintings of pale grids and horizontal bars or bands outlined in subtle pencil lines exhibit regular rhythm. So does Bridget Riley’s “optical” paintings that juxtapose contrastive colours arranged by serialized variations in size, shape, or placement in an all-over pattern.

The fact that entrainment is pervasive and mandatory makes it compelling to ascribe it an important role. For instance, it is known to cause stability, reliability, and predictability, and promote cooperation and feelings of familiarity and affiliation. On the other hand, this role will not be specific to aesthetic experience: Entrainment is involved in any kind of interaction, and therefore its explanatory value is comparatively small in the present case. Louwerse et al. (2012) remark that “pervasive synchrony is cognitively cheap but potentially useful across contexts and functions.” They suggest that entrainment is multifunctional and trades off during activities or processes of high complexity. What use might it have in the present context? Is there a trade-off?

I submit that by organizing and stabilizing the interaction of coupled systems, *entrainment creates an implicit common ground* that reduces uncertainty and provides the baseline for intentional explorative behaviour. On the present view, entrainment prepares for open-ended forms of interaction such as engagement that are available to conscious awareness but not necessarily cast in words.

## Moving together: the means for participatory sense-making

Aesthetic engagement constitutes the phenomenological side of coupling to an artwork and provides the context for moving, seeing, and feeling with art: It is where action, perception, and lived experience meet. Drawing on the fundamental similarity between aesthetic and second-person engagement, I suggest that research about the origin of empathy in dyadic interaction provides reason for giving movement a central place in the account of aesthetic experience.

According to developmental psychologist Peter Hobson, interpersonal engagement is characterized by jointness, a notion that echoes our definition of empathy as the immediate recognition of another person’s experience as distinct from your own. Hobson (2005:201) maintains that jointness “comes with being moved just enough to sense the psychological orientation of the other in oneself, but as the other’s.” Consider the infant’s experience of fear in response to the visual cliff. The visual cliff was designed to test depth perception, and consists of a sheet of Plexiglas that covers a cloth with a high-contrast draughtboard pattern (Gibson and Walk 1960). On one side the cloth is placed immediately beneath the Plexiglas, on the other it is dropped 4 feet below. The cliff is merely visual since the Plexiglas supports the infant’s weight. Hobson (2005) asserts that the infant’s experience of the visual cliff will change, if the infant can be made to respond to the caregiver’s feelings instead of its own. By enacting the caregiver’s feelings, the infant will be moved to occupy another stance in relation to the world without physically changing places and eventually crawl across the cliff. The experience of *emotionally moving* through somebody else makes for understanding that the world can be experienced in different ways and conversely, meaning can be known together.

By analogy, I claim that works of art can change the viewer’s perspective on the world by causing emotions and experiences in her that constitute other ways of responding to it than her own and thereby re-orient her. Swedish artist Lena Cronqvist’s two paintings of a young girl standing up and holding a doll in her left hand demonstrate how a superficially straight-forward naturalistic rendering of an everyday situation can move the viewer into an unsettling state of mind foreign to the normality of the situation (*Lilla flicka i röda skor med docka* and *Flicka med hand för munnen och docka*, both 1997; Castenfors and Fogelström 2014). A slight twisting of the representational conventions of naturalistic art such as perspective, shape, and colour and the conventional expectations about material daily life, e.g. the appearance of dolls, will cause experiences in the viewer that reflect another psychological orientation than her own.

*Bodily moving* occurs in participant perception and the co-enactment of behaviour, e.g. spoon-feeding when the father opens his mouth while approaching the spoon to the baby’s face in anticipation of the baby’s opening its mouth, and the baby then joins into the father’s action. The qualitative experience of seeing and feeling another agent’s movements moves the observer to match her own body movements to those of the other agent, which results in the observer’s being bodily moved through somebody else. Sometimes bodily moving together implies sharing the goal and, if successfully, reaching it together, as in the spoon-feeding example. Hence, an agent can participate in another agent’s attitudes and intentions by being (bodily) moved to move with her. The matching movement does not have to be an exact replica of the original: What matters is the mutually manifest, multimodal coordination of bodily orientation, intention, emotion, and attention that enables empathy and cognitive and affective perspective-taking.

I maintain that similarly to how joint movement allows parent and infant to recognize each other’s experiences and attitudes in dyadic engagement, it allows viewers to empathize with artworks in episodes of aesthetic engagement: Movement constitutes the source of aesthetic experience. This line of thought receives support from art educators, who tend to expose the inadequacy of discursive knowledge. They stress the importance of embodied learning to get in proper contact with art and develop an understanding that in a tangible way involves the viewer. Hubard (2007) provides several examples of embodied learning that each promotes active engagement as a manner of gaining a deeper understanding: replicating a form or content by impersonation; making sounds in response to visual stimuli; drawing the details of a sculpture, e.g. the lines of a hand; transforming paper, e.g. looking at a mandala and tearing, folding and forming the paper in correspondence to its features. Hubard’s examples link learning and experience to movement and intention, motion and emotion, and elucidate that bodily engagement with an artwork supports empathy and can lead to perceiving, acting, and feeling with it.

The remaining sections examine the relational dynamics between viewer and artwork from the two perspectives of bodily and emotional engagement. The common denominator is movement, which reflects the view that “[M]ovements are at the centre of mental activity: a sense-making agent’s movements—which include utterances—are the tools of her cognition” (De Jaegher and Di Paolo 2007).

## Bodily engagement: the perception–action loop

In a case study of dialogic looking in a gallery setting, McKay and Monteverde (2003) argue that aesthetic experience requires that subject and object are integral parts of each other and ends in both being transformed. The image of two equal players who mutually constitute or define each other is attractive and recalls the characterization of structurally coupled systems as mutually specifying each other. McKay and Monteverde conclude that engaging in an active and supervised dialogue with the artwork leads to a unique and unified perspective. Unfortunately, because they focus on the verbal aspects of dialogue—externally with other viewers and art educators, internally with the self, they by-pass the bodily, experiential, and emotional aspects of understanding.

Generally, we make sense of the world by physically moving around in it and discovering affordances for action and attune to variations in the environment by modifying and calibrating our perceptual expectations and motor actions. Because agency structures perception, locomotion in physical space will organize the perception of the environment in ways that correspond to current needs and afford novel actions (Yamamoto 2012). Those actions will cause other variations and eventually result in further specifications. In short, action specifies perception and perception specifies action.

By the same token, visitors engage with artworks in the exhibition room by moving around, circling the artwork, looking at it from a distance or close up, from below or the left or right, sitting down on the bench in the middle of the room or taking tours focusing on several items at a time and alternating gaze between them. Eventually they end up with a dynamic map of the exhibition tuned to their interests and needs.

Specifically, perceptual feedback from body movements made in response to the visual experience of an artwork will cause the viewer’s behaviour to change and so results in other visual experience, etc. This progressive dynamics constitutes the *perception*–*action loop* (*PAL*) *of bodily engagement*. It allows viewers to visually explore artworks by letting the artefact guide their movements through physical space, in agreement with the observation that an agent can participate in others’ attitudes and intentions by simply following them, *being* (*bodily*) *moved to move with* them, or by actively seeking to sense their orientation, (*bodily*) *moving to move with* them. The viewer makes sense of her actions in subjective or lived physical space comparing actual with anticipated outcome and as she discovers new routes through objective physical space.

Looking up close reveals detail while looking from the far end of the room lets you take in the entire artwork at a single moment, feeling its full force. Changing positions discloses new aspects of it, leading to further changes of position, and so on. Because perception is a function of movement and position in space and time, walking around or, on a smaller scale, moving in and out of postures and alternating body orientation will modify the viewer’s perception of the artwork substantially. Small variations in body position and movement can have significant effects on the perception of colour, size, height, width, texture, or grain. Thus, surfaces, illumination, and shadows determine how things look with respect to colour. Moving continuously changes the light conditions of visual experience and thereby also how the colour of a given object looks to the agent. As you move relative to an object you are observing, you encounter its visual potential by a series of aspects. Each of the agent’s movements and actions enact her experience of the artwork at the time of its performance.

Visual experience presents the world along two dimensions: egocentred route maps from the perceiver’s vantage point and allocentred survey maps from a disembodied position accessed inferentially (Morganti 2016). Morganti (2016:111) describes wayfinding as “a complex and continuously changing balance between the information available both in route and survey perspective”. In Morganti’s view, the agent’s surrounding space consists of the affordances that at present are available to her, and how things look to her is constrained by sensorimotor skill that reflects learning. Morganti’s research in spatial cognition suggests that the experience of a given artwork, say, one of the paintings from Claude Monet’s series of water lilies, will vary between agents and also within one and the same agent with respect to time.

To summarize the discussion so far, in addition to learning history, a viewer’s aesthetic experience will depend on how she is moving through the exhibition space, the movements she makes while doing so, and what parts of material space she cares to integrate into her spatial map along the way.

The more invitations to interact from artworks that a viewer responds to and the more ways of responding she masters, the more she will learn about her real possibilities to explore art visually and her ability to control the process. Perception partly is a socio-cultural skill, and so is motion. Aiming to explain how high-level cognitive processes arise from low-level perceptual and motor abilities, Hutchins (2010) argues that in culturally constructed settings, bodily motion can acquire meaning by virtue of its relation to the spatial organization of things that has developed in the past to scaffold behaviour. In our times, viewers often learn to interact with art by moving through exhibitions spaces. That physical context modulates the relation between aesthetic experience and viewing behaviour is attested by Brieber et al. (2014) who examined free viewing of an art exhibition in the context of either the museum or laboratory. The study reveals that participants in the museum context liked the artworks more, found them more interesting, and viewed them longer.

The exhibition space is a result of design, created with a certain purpose. Such designated areas of the shared environment come with a set of functional properties that afford specific activities (Gibson 1986). Barker’s (1968) notion of behaviour setting refers to a cohesive set of standing patterns of behaviour that together with their physical surroundings provide the spatial and temporal boundaries of an activity. Behaviour settings regulate and facilitate the performance of social activities, promoting their continued existence. To illustrate, an art gallery has walls, doors and windows, and within there are physical boundaries that divide the space into sections, e.g. passages where visitors can rest their senses, areas where they find information about the exhibition and the featured artists, larger spaces where the artworks are located, spots (vantage points) designated for observation of the individual pieces, an area close to the front door where visitors can compose their thoughts and make themselves ready to leave, etc.

The design of exhibition spaces encourages visitors to engage with the artworks, but provides limited assistance for sense-making—how much support visitors get is in the hands of the management and the curator. The artist’s part in this seems peripheral. Hautala (2015) strengthens this impression in describing how an artist takes breaks to walk around and view her artworks while hooking them in a museum, following the same routes that she expects the visitors will take, hanging the pieces accordingly. The option to move the walls or change routes is not mentioned. Separating the artist’s goal of achieving the artwork from the curator’s goal of placing it in an appropriate historical and theoretical context that respects tradition and praxis, Hautala claims that artworks find their final form by being assigned a location in the museum space, an address as it were.

## Emotional engagement: the motion–emotion loop

Proprioception refers to the sense of movement and position that includes tactility, gravitational orientation, force, and kinesthesis. Kinesthesis refers to the awareness of dynamic movement, a qualitatively felt kinetic flow that may be experienced as expansive, abrupt, weakened, jagged, curved, constricted, fast, etc. (Sheets-Johnstone 2010). Kinetic flow is interrelated with affect. Exploring how her experience in dance influences her educational research, Stinson (1995:44) addresses the intersubjectivity of kinesthetic sense, claiming that it

(…) heightens our awareness both of the other who is outside us and of what is inside ourselves. It allows us to notice what we are feeling in our own interior, letting us know when we are stiff or fatigued or upside down, whether our fingers are stretched apart or close together. The kinesthetic sense thus both tells us about ourselves and connects us with others as embodied selves.

Sheets-Johnstone (1999) argues that the function of emotion is to motivate action. Changes in body posture manifest the onset of emotion that determines the agent’s readiness to act, and exemplify the respect in which agents are “moved to move” (Fuchs and Koch 2014). The causal influence between emotion and action goes in both directions; motion (the process of moving) and emotion intrinsically connect (Sheets-Johnstone 1999). Hence body posture may have a global impact and trigger emotion. Furthermore, what may seem like minor behaviour can have major consequences, e.g. orientation movements performed relative to a target of action will affect the agent’s emotional reactions to the target and thereby action readiness. Orientation movements demonstrate that agents can be “moved by movement” (Fuchs and Koch 2014).

From a theoretical position, Fuchs and Koch (2014) argue that emotion results from the circular interaction between affective qualities in the environment and the agent’s sensations and movements and that the body charges both self-experience and environment with valences. Specifically, body feedback promotes the experience of emotion, formation of attitudes, and emotion and behaviour regulation. Koch (2014) examined the effects of dynamic body feedback from position and movement on affect and attitude, relating movement rhythm (changes in muscle tension and properties related to space, weight, and time) associated with smooth versus sharp reversals to movement shape (changes in the form or direction) in the form of approach versus avoidance motor behaviour. Movement rhythms were shown to influence affect and attitude and modulate the influence of movement shape on attitudes, e.g. smooth rhythms and approach movements cause more positive attitudes.

The attested interdependence and continuity between motion and emotion corroborate that aesthetic experience originate in perceived (in the artwork) or executed (with respect to the artwork) movement. This means that the viewer’s psychological reaction to an artwork depends on the movements that the artwork produces in her while she is looking at it. Bodily responses to works of art have movement qualities and shapes and cause changes in body posture that cue emotion. Emotion triggers approach or avoidance behaviour and determine the manner and direction in which the interaction between viewer and artwork proceeds. Hence, body feedback from movements made in response to works of art triggers an emotional response that modulates affect and attitude, which is to say that the viewer is emotionally moving with the artwork. The qualitative feel of movement makes the interaction intrinsically meaningful. Equally, the viewer’s psychological reaction may depend on the emotion that the artwork causes her to act out, as in the visual cliff. This makes the agent react to the displayed content by the induced experience.

I will refer to this dynamics as the progressive *motion*–*emotion loop* (*MEL*) *of emotional engagement*. *Being moved* (*emotionally*) *to movement or action* and *moving or acting to be moved* (*emotionally*) by a work of art both involve moving with the artwork, sensing its psychological orientation in oneself (cf. Hobson 2005).

Two works of art that exert strong effects on the viewer and have the quality to move people will illustrate the present line of thought. To begin, consider Yves Klein’s paintings in monochrome blue, or *the Blue Monochromes*, the first one made in 1957. They were painted with a roller in a pure blue pigment IKB International Klein Blue, the surface without any personal touch or marks. Then they were mounted in front of the wall, not on it, leaving them untouched by the forces of physical space. The intensity of the blue colour draws the viewer into the canvas and is intended to make her transcend the material painting and feel totally immersed in hue, not allowing her to find a fix point or centre of interest. Klein intended the boundary between artwork and viewer to dissolve completely, leading to a state of heightened sensibility.

The second example is Lucio Fontana’s *Concetto spaziale*, a collection of works begun in 1949 that consist in holes and slashes on the surface of monochrome paintings, the strongest impact being made by slashes on white, red and raw canvases. The slashes turn the two-dimensional work into a three-dimensional one and create depth where there is none. They lead the viewer’s gaze towards the holes in the canvas, and leaves her struggling to see what if anything is hiding in the gaps. The slashes are obviously manmade, brutal while precise, made with a sharp object and by determinate rhythmic movements that cause motor and emotional resonance in the viewer’s brain (Umilta et al. 2012). The gaps, like the slashes, can seem both intriguing and frightening, anticipating the unknown via the darkness looming below the surface of the canvas. Klein’s and Fontana’s works grab the viewer as it were both bodily and mentally. In playing with the experience of space, the paintings throw the viewer off balance, Klein’s by producing the illusion of free floating and Fontana’s because the cuts cause the perception of depth and dynamism, creating the illusion of reality where there is none.

## Concluding remarks

I have argued that non-discursive aesthetic experience emerges when the viewer engages with the artwork in physical and material space via the processes of bodily and emotional engagement. These processes permit the viewer to move with, be moved by, or move to be moved by the artwork, all of which promote perceiving, acting, and feeling with the artwork as in empathy and perspective-taking between human agents. Perception, action, movement, emotion, motion, and affect are inseparable elements of the relational dynamics.

I have described the interaction by two processes operating at different temporal and spatial scales, arguing that the perception–action loop organizes and structures visual experience by specifying it, while the emotion-motion loop generates qualitatively felt embodied meanings that modulate over-all affect and attitude. The distinction reflects the explanatory purpose of exposing the two basic dimensions or functions of aesthetic engagement. In practice the processing of aesthetic experience is not layered, but there is interaction not only within processes, but also between processes that succeed each other in time (horizontally) and occur simultaneously (vertically).

Aesthetic experience is based in the bodily experience of motion and direction and has an inevitable affective and evaluative dimension. The experience of affect supports on-line evaluation of the sense-making process as the viewer continually adjusts her body movements to maintain interaction while moving in and out of synch. Movement and motion are value-laden and condition the viewer’s sensory experiences and feelings for the artwork and the interaction as a whole, and therefore influence actions and behaviour that unfold on the larger, intermediate temporal and spatial scales to which people usually direct their conscious attention, for instance, when they as visitors circle the museum space to find the optimal vantage points for taking in the individual artworks currently on display one by one. By bodily and mentally moving with the artwork viewers can actively exploit material space for exploration and seek out positions and trajectories that are conducive to making sense.

We can think of engaging with an artwork as a second-person relation characterized by openness and curiosity, making way for understanding. As the interaction between viewer and artwork unfolds, the agent will notice new aspects of the artwork and new patterns of variations will emerge that increase the complexity and saturation of the interaction.

The view that bodily movement is essential to aesthetic experience reflects the conception of the visual arts practices as enactive and literally making things visible. According to the German painter Paul Klee (Gale 2013) art does not reproduce the visible; rather, it makes visible. Works of art consequently may provide guidance for seeing and knowing to artist and viewer alike.
